# Preparation and Characterization of a Novel Poly(vinylidene fluoride-co-hexafluoropropylene)/Poly(ethersulfone) Blend Membrane Fabricated Using an Innovative Method of Mixing Electrospinning and Phase Inversion

**DOI:** 10.3390/polym13050790

**Published:** 2021-03-04

**Authors:** Norhan Nady, Noha Salem, Sherif. H. Kandil

**Affiliations:** 1 Polymeric Materials Research Department, City of Scientific Research and Technological Applications (SRTA-City), New Borg El-Arab City, Alexandria 21934, Egypt; 2 Department of Materials Science, Institute of Graduate Studies and Research, Alexandria University, Alexandria 21526, Egypt; nohasalem560@yahoo.com (N.S.); s.kandil@usa.net (S.H.K.)

**Keywords:** poly(vinylidene fluoride-co-hexafluoropropylene), poly(etherrsulfone), membrane distillation, novel blend membrane, electrospinning and phase inversion mixed techniques

## Abstract

In this work, a novel polymeric membrane was innovated in terms of composition and preparation techniques. A blend of poly(vinylidene fluoride-co-hexafluoropropylene) (PcH) and poly(ethersulfone) (PES) (18 wt.% total polymer concentration) was prepared using a N-methylpyrrolidone (NMP) and N, N-Dimethylformamide (DMF) solvents mixture, while Lithium chloride (0.05–0.5 wt.%) was used as an additive. The electrospinning and phase inversion techniques were used together to obtain a novel membrane structure. The prepared membranes were characterized using scanning electron microscope imaging, energy dispersive X-Ray, differential scanning calorimeter, thermogravimetric analysis, and Fourier transfer infrared spectroscopy-attenuated total reflectance analyses. Moreover, the static water contact angle, membrane thickness, porosity, surface roughness as well as water vapor permeability were determined. ImageJ software was used to estimate the average fiber diameter. Additionally, the effect of the change of PcH concentration and coagulation bath temperature on the properties of the fabricated membrane was studied. The novel developed membrane has shown a good efficiency in terms of properties and features, as a membrane suitable for membrane distillation (MD); a high porosity (84.4% ± 0.6), hydrophobic surface (136.39° ± 3.1 static water contact angle), and a water vapor permeability of around 4.37 × 10^−5^ g·m/m^2^·day·Pa were obtained. The prepared membrane can be compared to the MD membranes commercially available in terms of properties and economic value.

## 1. Introduction

The worldwide demand for freshwater for agricultural, industrial, and domestic applications is predicted to reach about 6900 billion m^3^/day by the year 2030, which would surpass the present available freshwater resources by about 40% [1,2]. Accordingly, researchers aim to find alternative clean water sources. The desalination of surface (brackish and sea) waters has been targeted as the primary alternative source for freshwater. Conventional desalination techniques could be classified into thermal techniques and membrane-based techniques. Thermal techniques usually employ heat to increase the water temperature above the boiling point and to transform it into a vapor that can be transferred and condensed in another region of the plant, which include multi-stage flashing (MSF) [3], multi-effect distillation (MED) [4], and mechanical vapor compression evaporation (VC) [5]. On the other hand, conventional membrane-based techniques usually incorporate the application of an external force to drive only the water particles through barrier (membrane) pores, thus trapping the salt on one side and keeping the other side salt-free. The most commonly used membrane-based desalination techniques include reverse osmosis (RO) [6], electrodialysis (ED) [7], and, recently, membrane distillation (MD) [8]. The ideal MD process needs the presence of the following criteria in the used membranes: high water contact angle (high hydrophobicity/non-wettability) [9], high porosity [10], small tortuosity factor [11], narrow and uniform pore size distribution [12], high liquid entry pressure of water (LEP, the pressure threshold at which liquid water would penetrate into pores to cause pore wetting of membranes) [10], thin thickness [13], low thermal conductivity (which would help reduce heat loss by conduction), good thermal and chemical stability [14], high resistance to fouling [15], and good mechanical strength [13,16,17]. These key factors are closely related to the membrane structure and morphology [18,19]. Various approaches have been investigated, with certain success, to optimize the membrane morphology and performance to be suitable for MD, including investigating new polymers [20] and/or the surface modification [21] of existing polymers by different techniques such as blending [22,23], compositing [24], and the incorporation of nano-additives [11,25,26].

There are various methodologies that are feasible for the fabrication of membranes for MD applications, including electrospinning [27], phase inversion [28], supercritical assisted phase inversion [29], and mechanical stretching methods [25]. Many polymers, such as polypropylene (PP) [30], polyethylene (PE) [31], polytetrafluoroethylene (PTFE) [32], poly(vinylidene fluoride) (PVDF) [33], polysulfone [34], and polydimethylsiloxane [35], are well-known materials for MD applications due to their good chemical resistance, thermal stability, and low thermal conductivity [36,37,38,39]. However, there are many drawbacks that make these materials practically unsatisfactory for MD applications. For example, PVDF is chemically inert and possesses no surface groups, so that any surface modification to the membrane surface is difficult. Moreover, PVDF membranes have low surface tension substances and are therefore prone to wetting [40]. PP membranes are susceptible to wetting and fouling [41]. PTFE membranes have poor compatibility with other polymers [36], and their processing is relatively difficult [42]. Hence, it is necessary to develop a new membrane with high performance and reasonably priced as one of the major challenges in the MD process [43].

Poly(ethersulfone) (PES) is a low-price commercial polymer membrane that can be easily fabricated by the phase inversion method and is widely used in separation processes due to its high thermo-plasticity, excellent mechanical properties, good chemical and thermal stability, and relatively higher transition temperature. However, it is not the preferable membrane material for MD applications because of its limited hydrophobic characteristics [44]. Therefore, membrane modification is required for PES to meet MD process requirements. For example, titanium oxide nanotubes were incorporated into PES, this fabricated membrane showed high salt rejection (98%), with a permeate flux that reached 15.2 kg/m^3^ when it was applied to the desalination of 7000 ppm feed salt concentration by the VMD process [45]. In other work, where PES was blended with ethylene glycol (EG) and PVDF, the PVDF-EG-PES membranes exhibited improved water permeability, an average flux of 15.3 kg/m^2^·h, and NaCl rejection maintained at between 99.9 and 99.3% throughout the 20 h experimental period using a direct contact MD system [46]. Poly(vinylidene fluoride-co-hexafluoropropylene) (PVDF-HFP or PcH) is a recently developed co-polymer [47]. PcH is prepared by the non-solvent induced phase separation technique, and it has many advantages which make it a promising membrane material to be used with PES membrane matrix including high hydrophobicity, high solubility, low crystallinity, small glass transition temperature, and great free volume [48,49,50,51]. Recently, a novel triple-layer nanocomposite membrane was developed by the electrospinning method. The membrane was fabricated using PES/carbon nanotubes (CNTs) as the primary bulk material and PcH/CNTs as the outer and inner surfaces of the membrane. The developed membrane showed a high performance in the DCMD system in terms of membrane porosity (91 ± 1.8%), membrane hydrophobicity (144° ± 2° static water contact angle), permeation flux (22.2 kg/m^2^·h water flux using 10,000 ppm feed concentration), and salt rejection (99.9%) [27]. However, the weak physical cohesion forces between the layers are still considered a deficiency of the cohesion of the membrane, which be handled with great care during the installation of the MD unit. Moreover, the use of the mixing method using the lower percentage of PES with the greater proportion of PcH was not economically feasible and was not sufficient to obtain the required characteristics of MD membranes.

In the current study, a novel membrane is developed. The new membrane was prepared by blending PcH with PES using a specific solvent mixture and Lithium chloride as an additive. The new membranes were prepared using two techniques: electrospinning followed by heat treatment (EH technique) and by using an innovative way, which is electrospinning followed by phase inversion (EPI technique) to obtain a novel membrane structure. The prepared membranes were fabricated and characterized using different analysis techniques, such as scanning electron microscope (SEM) imaging, Energy Dispersive X-ray (EDX), differential scanning calorimeter (DSC), Thermogravimetric analysis (TGA), and Fourier transfer infrared spectroscopy-Attenuated total reflectance analyses (FTIR-ATR). Moreover, the static water contact angle, membrane thickness, surface roughness, membrane porosity as well as water vapor permeability were determined. ImageJ software was used to estimate the average fiber diameter. The effect of the change of the PcH concentration and coagulation bath temperature was highlighted.

## 2. Materials and Methods

### 2.1. Materials

**Figure 1:**
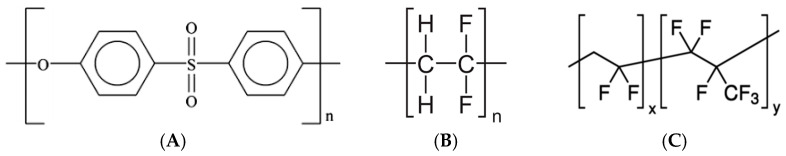
The structure of (**A**) Poly(ethersulfone) (PES), (**B**) Poly(vinlidine fluoride) (PVDF), and (**C**) Poly(vinlidine fluoride-co-hexafluoropropylene) (PcH) polymers.

Poly(ethersulfone) (PES) Ultrason E 6020P (glass transition temperature T_g_ = 225 °C and a molecular weight (M_w_) of 58,000 g/mol (polymer density = 1.37 g/cm^3^) was obtained from BASF chemical company (Ludwigshafen, Germany). Poly(vinlidine fluoride-co-hexafluoropropylene) (PcH) copolymer pellets (density 1.78 g/cm^3^ at 25 °C, T_m_ is around 135–140 °C according to ASTM D3418, viscosity is in the range of 20,000–25,000 poise (230 °C) (100 s^−1^L), Mw 214.06 g/mol), N,N-Dimethylformamide (DMF) (HPLC, 99.8%, density of 1.4305 g/mL, Mw = 73.09 g/mol, viscosity of 0.796 cp at 25 °C, polarity index of 6.4, dielectric constant of 36.7, 153 °C boiling point, and surface tension of 35.1 at 20 °C in mN/m), and N-methylpyrrolidone (NMP) (>99% purity, density = 1.028 g/cm^3^, Mw = 99.13 g/mol, viscosity of 1.65 cp at 25 °C, polarity index of 6.7, and dielectric constant of 32.2, 202 °C boiling point, and surface tension of 40.8 at 20 °C in mN/m) were purchased from Sigma-Aldrich. Figure 1 illustrates the chemical structure of the PES (A), Poly(vinlidine fluoride) (PVDF) (B), and Poly(vinlidine fluoride-co-hexafluoropropylene) (PcH) (C) polymers.

### 2.2. Methods

#### 2.2.1. Membrane Dope Preparation

The pure PES and pure PcH dope were prepared by mixing 18 wt.% polymers using a 1 NMP:9 DMF solvents mix until fully dissolved, which resulted in a viscous, homogeneous solution. The blend membranes were prepared by using the PcH/PES blend polymers using pure NMP, pure DMF, and the 1 NMP:9 DMF solvents mixture. A specific amount of Lithium chloride was added. The dope was stirred for 12 h at 150 rpm at 60 °C. Then, the prepared dope was degassed and fed to a syringe before starting the electrospinning process.

#### 2.2.2. Membrane Formation

**Figure 2:**
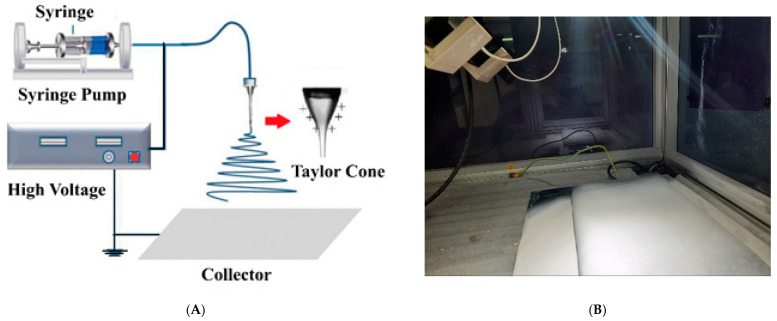
(**A**) A schematic diagram of the electrospinning process [26] and (**B**) A photo of the fabricated membrane before the post-treatment.

The solution was drawn into a 10 mL syringe that was placed in a syringe pump for electrospinning. Electrospinning was performed using (nano-01, MECC CO., LTD. JAPAN, Fukudo, Ogori-shi, Fukuoka, Japan). Figure 2 shows a schematic diagram of the electrospinning process and a photo of the fabricated membrane before the post-treatment. The solution was first fed to the syringe with a stainless-steel needle (12.3 mm inner diameter, OD: 0.9 mm, ID: 0.6 mm, MECC CO, Fukudo, Ogori-shi, Fukuoka, Japan). The distance between the needle tip and the collector was kept at 17 cm, and an electric voltage of 25–28 kV was applied with a spinneret speed and widths of 50 mm/s and 120 mm, respectively. The flow rate of the polymeric solutions was 1.5–2.0 cc/h, and the electrospinning time was 5 h. The home-made collector was used; the collector was a flat carton sheet covered by aluminum foil, and, on top of it, a plastic net support was fixed. After completion of the electrospinning process, the membrane was cut into two parts: one part was kept in the oven at 60 °C for 24 h, and the second part was immersed in distilled water at a specific temperature for 2 h. 

Table 1 presents the composition and the coded name of the fabricated membranes. The one-factor-at-a-time approach was applied; pure solvent, pure polymer, LiCl concentration (0.05, 0.1, or 0.5 wt.%), PcH concentration (4, 8, or 12 wt.%), and coagulation bath temperature (40, 60, or 80 °C) were studied.

#### 2.2.3. Membrane Characterization

The porosity (***ε***) of the fabricated membranes was obtained by measuring the wet and dry weights of the membrane samples. The wet weight of the membrane sample was measured after immersing it in absolute ethanol for 15 min. After drying the sample using an oven at 60 °C for 24 h, the dry weight of the sample was measured. The membrane porosity was determined using the following equation [50]:

(1) $$\varepsilon =\;\frac{\frac{m_{w}-m_{d}}{\rho_{e}}}{\left[\frac{m_{w}-m_{d}}{\rho_{e}}\right]+\frac{m_{d}}{\rho_{P}}}$$

where $m_{w}\;$ is the wet membrane weight (g), $m_{d}\;$ is the dry membrane weight (g), $\rho_{e}$ is the density of ethanol (g/cm^3^), and $\rho_{P}$ is the density of the polymer or polymer blend (g/cm^3^). SEM images were used to determine the average fiber diameter of the prepared membranes.

The static water contact angle for membrane samples was measured using the Gonimeter model 500-F1 coupled with a video camera and image analysis software. A water droplet of 7 µL was dropped on different spots of the membrane surface. The membrane sample was analyzed using the captured images at consecutive time frames, the right and left contact angles were estimated using the image analysis software, and the mean value was determined. The reported value was the average of eight readings on three different samples for each membrane.

GBI W303 (B) Water Vapor Permeability Analyzer (Labthink Instruments Co., Ltd., Jinan, China) was used for the evaluation of the water vapor transmission rate (WVTR) via the cup method. The water vapor transmission rate was calculated by measuring the amounts of water vapor transferred through a unit area of the film in a unit time below the exact conditions of temperature (38 °C) and humidity (4%) as stated by the subsequent standards (ASTM E96). Once the WVTR was determined, the water vapor permeability coefficient was calculated according to the following equation:

WVP = WVTR × L/PWVS (RH1 − RH2)(2)

where WVP is the water vapor permeability coefficient (g water. mm m^−2^. day^−1^. mm Pa), WVTR is the water vapor transmission rate through a film (g water. m^−2^ day^−1^), L is the mean film thickness (μm), and PWVS is the partial water vapor saturation pressure at the test temperature (mmHg). RH1 is the relative humidity of the chamber (0.75), and RH2 is the relative humidity inside the capsule with desiccant (0% RH).

The average membrane thickness was calculated as an average of eight measurements at different points on three different membrane samples using a digital micrometer (range: 0–25 mm, precision: 2 µm, HDT, China).

The average roughness of prepared membranes was tested using a surface roughness tester (SJ-201 P, Mitutoyo, Kawasaki, Kanagawa, Japan). The membrane surfaces were fixed onto a glass plate before measuring. The calibration of the instrument was done by measuring the roughness of the used glass plate. The recorded results are the average of eight measurements of three different membrane samples.

An FTIR-ATR analysis of the fabricated membranes was conducted using an FT-IR spectrophotometer (Shimadzu FTIR-8400S, Nishinokyo Kuwabara-cho, Nakagyo-ku, Kyoto, Japan) equipped with an ATR accessory in the range from 400 to 1800 cm^−1^. The samples were used after complete drying in air for 48 h.

The glass transition temperature (T_g_) of the PES polymer and the melting temperature T_m_ of the PcH polymer were measured with differential scanning calorimetry (DSC, NETZSCH, Gebrüder-Netzsch-Straße 19, DSC-200PC) at a heating rate of 10 °C/min. The samples were scanned over a temperature range from a room temperature of 25 to 500 °C under 30 mL/min nitrogen flow. The T_g_ was defined as the onset temperature of the change in heat capacity during the heating cycle.

Thermal gravimetric studies of the membranes were carried out using a thermal gravimetric analyzer (Shimadzu TGA-50, Nishinokyo Kuwabara-cho, Nakagyo-ku, Kyoto, Japan). The samples were scanned over a temperature range from a room temperature of 25 to 1000 °C at a temperature gradient of 10 °C/min under 40 mL/min nitrogen flow.

Scanning electron microscopy (SEM, Joel Jsm 6360LA, Akishima, Japan) was used to investigate the membrane morphology. The membrane samples were cut using a very sharp shaving blade and were then coated with gold and imaged at a voltage of 15/20 kV and a resolution of 1280 × 960 pixels. For cross-section imaging; the membrane samples were immersed in liquid nitrogen and were fractured to be processed for gold coating before imaging.

## 3. Results and Discussion

### 3.1. Solvents Mixture and Electrospinning Process

In the electrospinning process, the solvent evaporation rate and the polymer drying time depend on the solvent properties. It is critical for a successful process to select an appropriate solvent system. N-Methylpyrrolidinone (NMP) and dimethylformamide (DMF) were used in this work. Both of them are polar aprotic and have low viscosity. They are miscible with water at all temperatures. NMP and DMF are good solvents with similar properties, such as polarity index and dielectric constant. The difference in the boiling point and surface tension of both used solvents [51,52] may affect the structure/shape of the formed membranes, as illustrated in the following points.

The polymer and solvent compatibility can be attributed to the difference in the overall solubility parameter. The compounds that have a large difference in solubility parameter are immiscible with each other. The total solubility parameter for poly(ethersulfone) (PES), the poly(vinylidene fluoride-co-hexafluoropropylene) (PcH), DMF, NMP, and water are 24.2, 23.2, 24.8, 23.1, 47.9 (calories/cm^3^)^1/2^ [53]. So, the overall solubility parameter of PES is much closer to DMF, and the overall solubility parameter of PcH is much closer to NMP. Thus it shows that the polymer mixture has a strong affinity with the used solvents. Therefore, the difference in the boiling points between the two solvents may affect the relative evaporation rate of each solvent and consequently the ratio of the presence of the (polymer) components on the membrane surface. The incomplete instantaneous formation of the polymer fibers and the droplet formation of the polymer blend (by gravity) on the support may be an indication of the incomplete evaporation of the NMP solvent. Another crucial parameter that affects the formation of electrospun fibers is surface tension. A decrease in surface tension leads to the establishment of electrospun fibers’ stocking beads. In other words, the higher the surface tension of a solution, the more it obstructs the electrospinning process owing to the unstable jets and the generation of sprayed droplets. For that, the pure NMP solvent badly affects the electrospinning process. The electrospinning process was much better in both pure DMF and the NMP/DMF mixture with a ratio of 1:9. Furthermore, the higher the value of the dielectric constant point of the chosen solvents affects positively on the electrospinning process, in which self-repelling charges on the surface of the polymer jet are sufficient to increase the Columbic stretching force, resulting in a strong elongation force with the formation of homogeneous free-beads fibers [54].

### 3.2. Membrane Hydrophobicity

**Figure 3:**
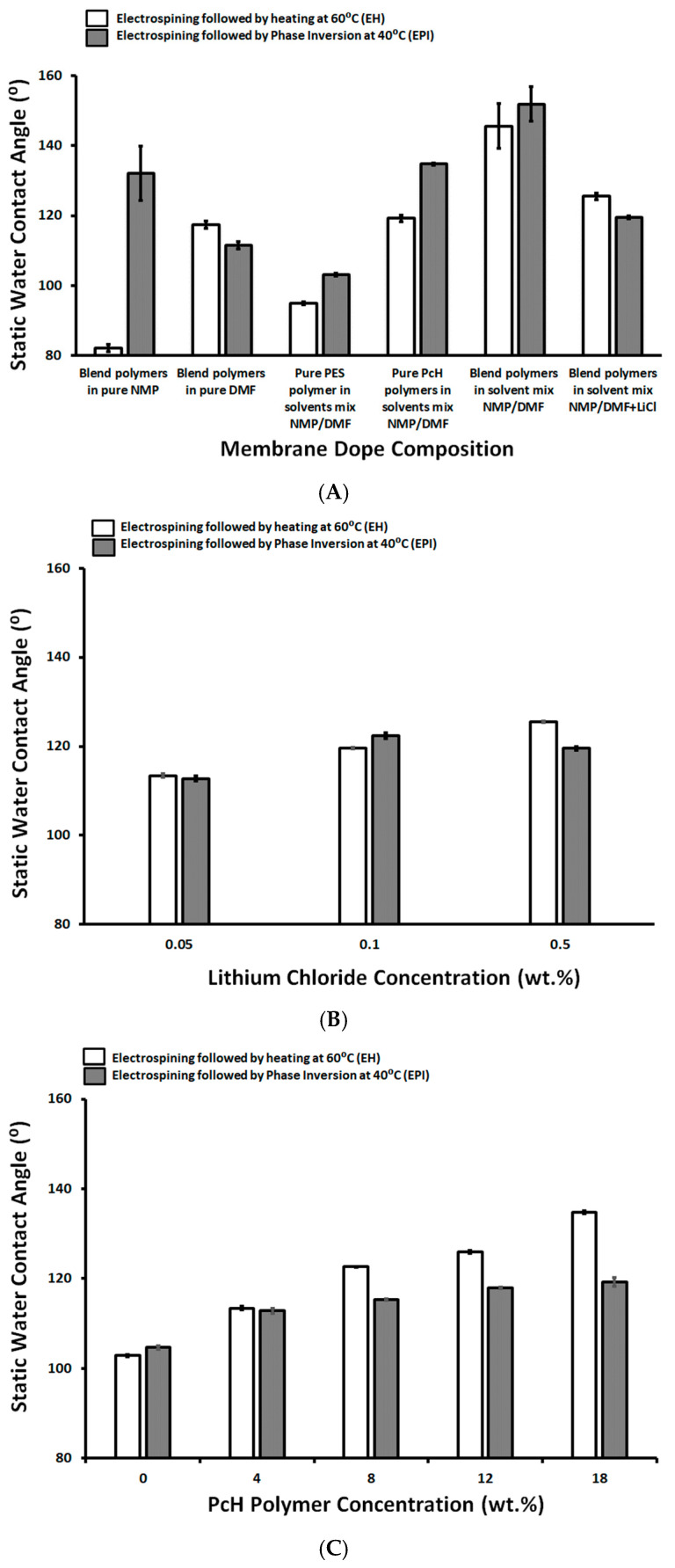
Static Water contact angle of different membrane dopes (**A**), as a function of Lithium chloride concentration (wt.%; **B**) and as a function of PcH polymer concentration in the membrane dope (wt.%; **C**).

The hydrophilicity of the prepared pure PES, pure PcH, and blended membranes was measured using the static water contact angle and was presented in Figure 3A. The presence of NMP traces on the membrane surface may affect the measured contact angle, as it was illustrated recently that the NMP concentration is dependent on the water contact angle of NMP/water mixed droplets on the PVDF sheet [54,55]. This previous work reported a contact angle of around 81°, that is very close to the determined contact angle (82.2°) in this work, of a blend polymer prepared by using pure NMP solvent followed by drying at 60 °C. This value increased to around 132.2° after the complete removal of the NMP solvent using the phase inversion post-treatment immediately after the electrospinning process [53,55]. As shown in Figure 3, the lowest measured static water contact angle value (95° ± 0.49) was measured for pure PES prepared using a 1 NMP:9 DMF solvents mix and post-treated by heating at 60 °C, while the highest static water contact angle value (151.8° ± 4.49) was measured for 14 wt.% PES/4 wt.% PcH blend membrane. This membrane was prepared using a 1 NMP/9 DMF solvents mix and post-treated by phase inversion at 40°C and without LiCl additive, which showed a beads-rich membrane microstructure as described in the following sections, that is associated with the thick (0.13 µm) and low porosity (68.4% ± 5.7) membrane.

The effect of the concentration of LiCl on the static water contact angle of the prepared membranes using both preparation techniques, heat treatment and phase inversion post step, was not significant—around 120 °C, as shown in Figure 3B. However, the increases of the LiCl concentration affected the electrospinning process and initiated the growth of the fibers in 3-D form and the formation of a horizontal lump of fibers. For that reason, the lowest concentration of LiCl was used to complete this work. As shown in Figure 3C, the increases of PcH concentration resulted in an increase in the measured static water contact angle. The increase in the measured contact angle was more pronounced in the case of post-treatment by heating the membrane at 60 °C than in the case of the phase-inversed post-treated membrane. This can be attributed to the incomplete removal of NMP solvent that keeps more PcH polymer on the membrane surface with a higher measured static water contact angle than the corresponding membranes treated by phase inversion. The increase in the coagulation bath temperature from 40 to 60 °C resulted in an increase in the measured static water contact angle (112.8° ± 0.06 and 136.39° ± 3.1, respectively) in a combination with very slightly decreases (3.1% reduction) in the determined membrane porosity and decreases in the measured membrane surface roughness (15.79% reduction relative to the surface roughness of membrane coagulated at 40 °C). However, more increases in the coagulation bath to 80 °C showed decreases in the measured static water contact angle to around 116.39° ± 2.1, an increase of the membrane surface roughness (12.5% reduction relative to the surface roughness of membrane coagulated at 40 °C), and a significant decrease in the membrane porosity (23.3% reduction). This behavior can be attributed to the co-effect of the rate of solvents evaporation, the coagulation water entry, and its syringe effect on the porosity and surface roughness of the produced membranes.

### 3.3. Membrane Porosity

**Figure 4:**
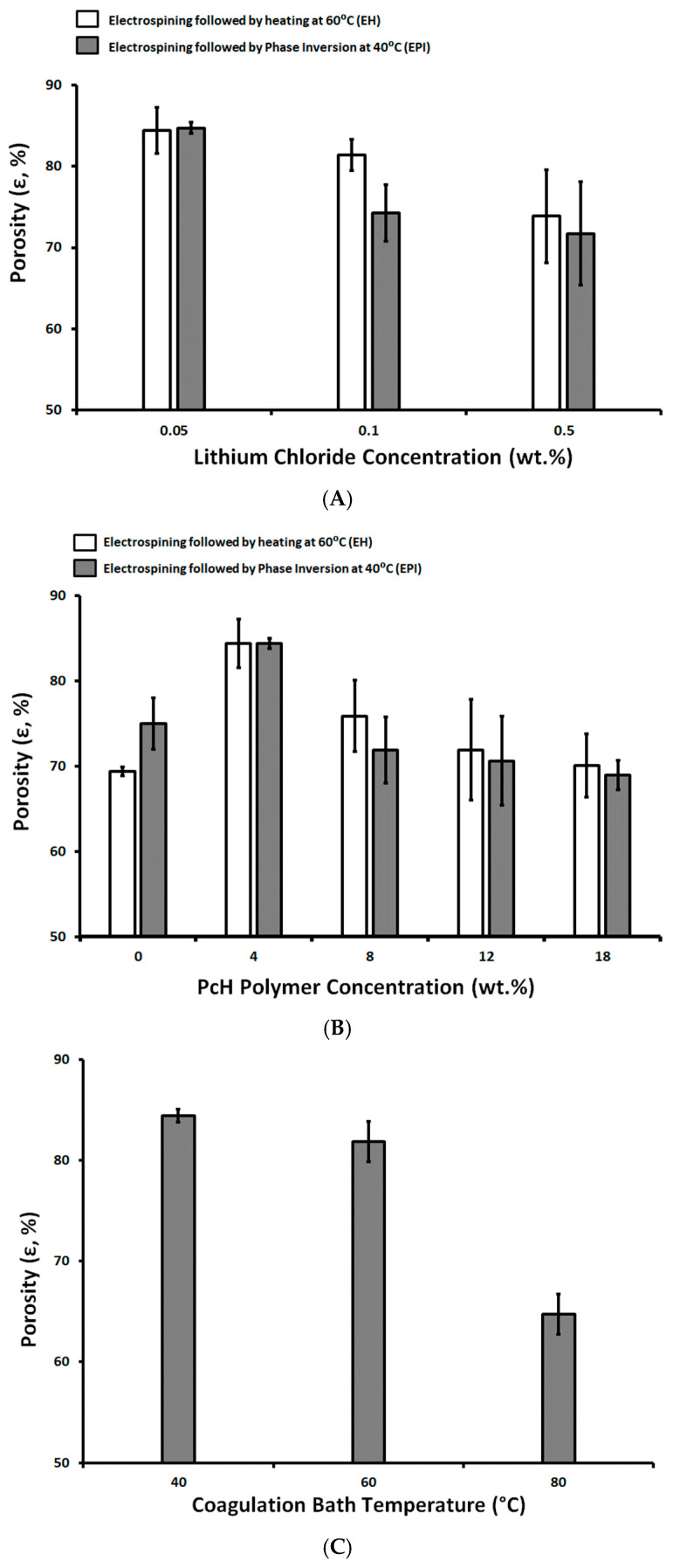
Membrane porosity as a function of Lithium chloride concentration (wt.%; **A**), PcH polymer concentration (wt.%; **B**), and coagulation bath temperature (°C; **C**).

Generally, the porosity of the membranes used for MD ranges between 30 and 85% [17]; however, the higher the porosity the larger the area for evaporation and the lower the thermal conductive losses of the membrane. As shown in Figure 4, the prepared membranes show high degrees of porosity, between 85.7 ± 2.7% and 85.9 ± 2.6%, for pure PES membranes prepared using a 1 NMP/9 DMF solvents mix and post-treated by drying at 60 °C and with a phase inversion at 40 °C, respectively. The high degree of porosity can be attributed to the reduction of polymer chain mobility and prevent the polymer from occupying the free space left by the evaporated solvents mix [56].

Three-dimensional fibriform network morphologies were formed in the case of the post-treated membrane by phase inversion at 40 °C. The pure PcH has a porosity around 70.1 ± 3.7 and 69.0 ± 1.7% for the EH and EPI preparation techniques, respectively, whereas the pure PES has 69.4 ± 0.5 and 75.0 ± 3.0% for the EH and EPI preparation techniques, respectively.

The increases in LiCl concentration seemed to affect the membrane porosity negatively. This effect can be attributed to an increase of solution conductivity, which consequently creates a smaller fiber diameter [53,54], suppresses the Rayleigh instability, and increases the bending instability of the produced fibers [57], which causes fibers to be randomly oriented and nonaligned. This facilitates the formation of 3D fiber lumps.

Adding PcH polymer to PES by 4 wt.% resulted in an increase in the membrane porosity. This can be attributed to the formation of a less viscous membrane dope than the pure PES that facilitates the demixing process and the higher porosity was determined [58]. The viscosity of a polymer solution has a significant impact on the mass transfer and heat transfer in the nascent membrane. The higher the increase of the PcH polymer’s concentration, the higher the membrane dope viscosity and the lower the rate of the solvent and non-solvent exchange, resulting in a lower rate of phase inversion and a lower porosity [59].

The increase in the coagulation bath temperature from 40 to 60 °C resulted in a very slight decrease (3.1% reduction) in the determined membrane porosity, but increased the coagulation bath to 80°C, showing a significant decrease in the membrane porosity (23.3% reduction).

### 3.4. Membrane Roughness

The surface roughness of the membranes has an important effect on the membrane characteristics; it is commonly known that the roughness parameter is linked to the contact angle of the membrane and its wettability [60]. In other words, surface roughening tends to increase the measure of the static water contact angle. Both Ra (arithmetic average of the roughness profile) and RMS are used for assigning surface roughness; Ra is calculated as the average roughness of surface-measured microscopic peaks and valleys, whereas RMS is described as the Root Mean Square of surface-measured microscopic peaks and valleys.

**Figure 5:**
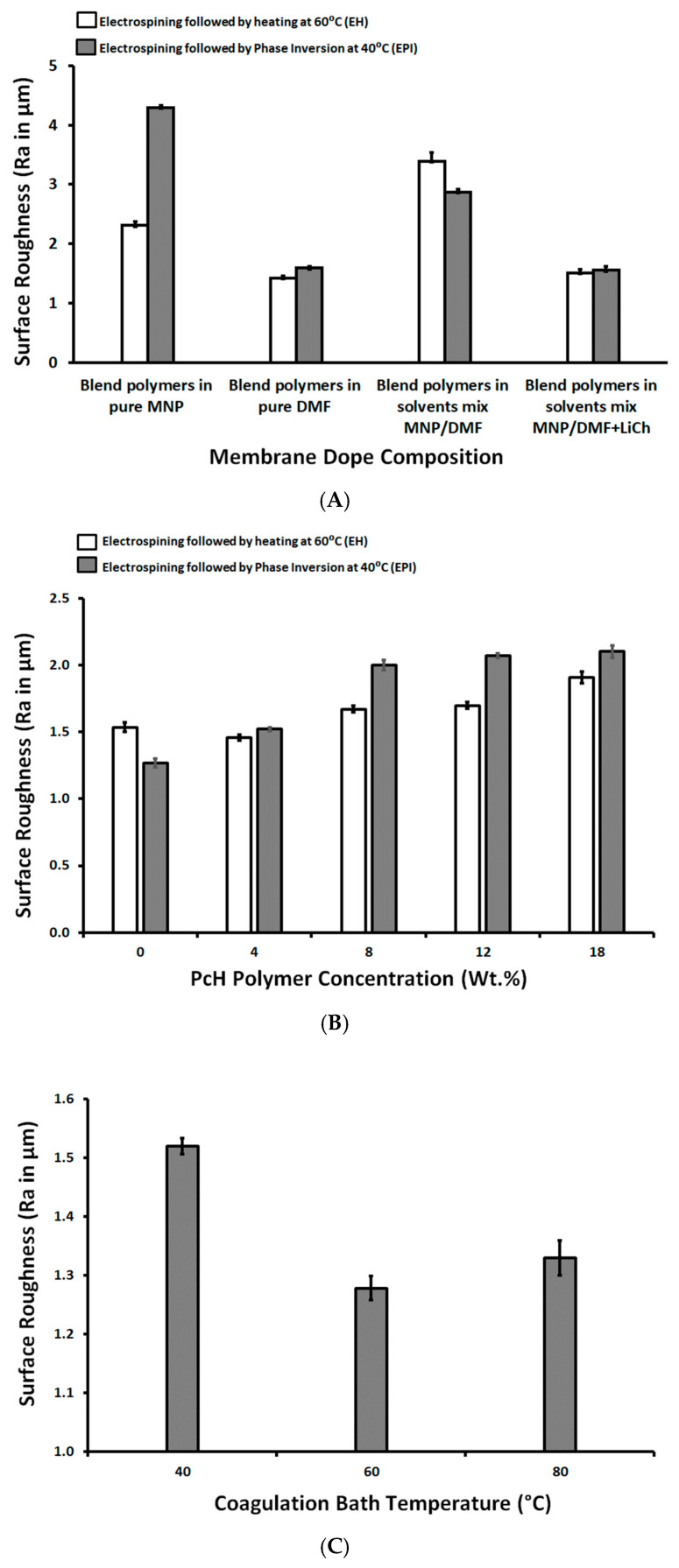
Surface roughness as a function of membrane dope composition (wt.%; **A**), as a function of PcH polymer concentration in the membrane dope (wt.%; **B**), and as a function of coagulation bath temperature (°C; **C**).

As shown in Figure 5, the lowest measured roughness (1.27 ± 0.03 µm) referred to the 14 wt.% PES/4 wt.% PcH blend membrane using a NMP/DMF solvents mix and post-treated by phase inversion at 40 °C and with a 0.05 wt.% LiCl additive, while the highest measured roughness (4.29 ± 0.04 µm) was measured for the 14 wt.% PES/4 wt.% PcH blend membrane using pure NMP solvent and without LiCl additive.

### 3.5. Membrane Thickness

**Figure 6:**
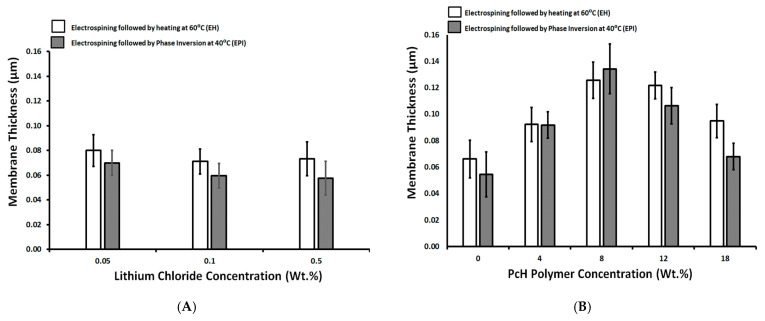
Surface roughness as a function of LiCl concentration (wt.%; **A**) and as a function of PcH polymer concentration in the membrane dope (wt.%; **B**).

The thickness of the prepared membranes was in the range of 0.04–0.14 µm (see Figure 6). As shown in Figure 6A, the concentration of LiCl had no significant effect on the formed membrane thickness with an average thickness range of 0.07 µm. The phase inversion post-treatment resulted in a slightly lower measured thickness average, of around 0.06 µm. This reduction in the thickness can be attributed to removing the residual solvents that may swell the polymer fibers, with a slight increase in the measured thickness in the case of a post-treated membrane by heating at 60 °C.

It is known from the literature [61] that the membrane thickness usually decreases with the increasing of polymer viscosity (assigned by the polymer molecular weight). However, the membrane thickness increases with an increase in the PcH polymer concentration up to 8 wt.%, and then the effect is inversed with a higher PcH polymer concentration, as shown in Figure 6B. This unspecified trend may be attributed to the formation of a blend of the PES and PcH with unexpected synergic effects on the membrane’s physical structure. On the other hand, this is probably due to the presence of the plastic net support, whose structure is well filled by the polymers and thus affects the thickness of the formed membranes. The thicker membrane was obtained with a composition of 14 wt.% PES and 4 wt.% PcH, without LiCl additive, which produced a completely different membrane morphology (beads-rich structure).

### 3.6. Membrane Microstructure

**Figure 7:**
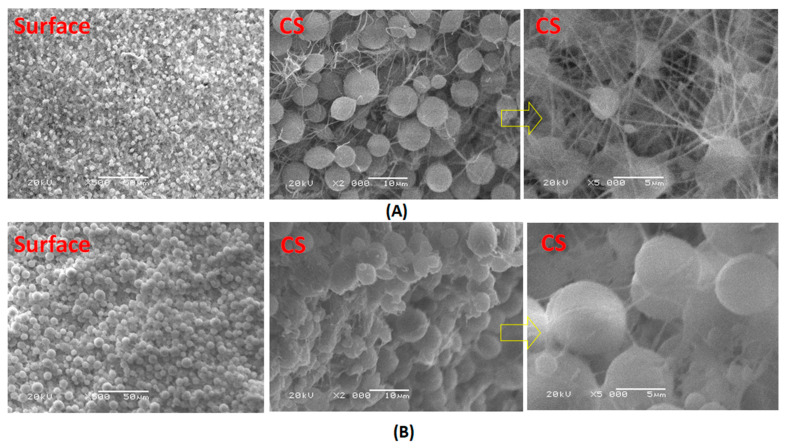
SEM images of PcH/PES blend membranes that were prepared using a 1 NMP: 9 DMF solvents mix and were treated by (**A**) heating at 60 °C and (**B**) phase inversed at 40 °C without LiCl additive. Surface images at ×500 and cross section images at ×2000 and ×5000 were done.

Figure 7 shows that the particulate morphology is the spherulitic structure characteristic of PVDF. The porosity of the membranes without LiCl additive increased up to ~85 in the case of the post-treated by heating at 60 °C. The formed compact morphology in the case of the post-treated by phase inversion at 40 °C associated with increased roughness, which related to the large spherulite morphology. The formation of the beaded nanofibers can be attributed to the low dielectric constant, high surface tension, and low viscosity of the membrane dope.

**Figure 8:**
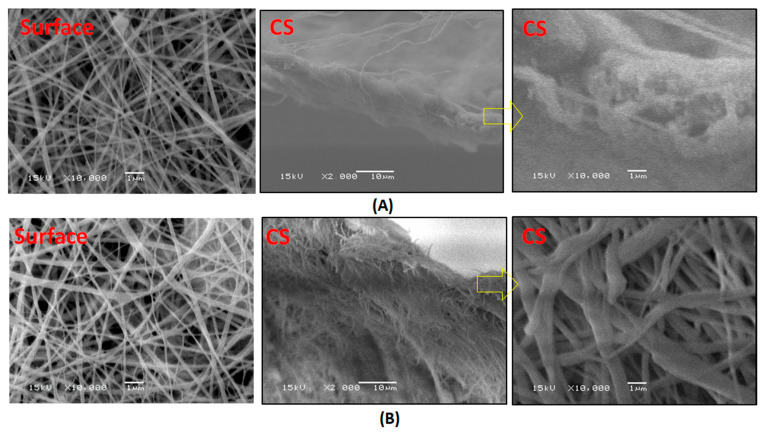
SEM images of PcH/PES blend membranes that were prepared using a 1 NMP: 9 DMF solvents mix and were treated by (**A**) heating at 60 °C and (**B**) phase inversed at 40 °C with LiCl additive. Surface images at ×10,000 and cross section images at ×2000 and ×10,000 were done.

Adding LiCl to the blend polymers enhanced the electrospinning process and resulted in free-bead fiber formation, as shown in Figure 8. The effect of LiCl is related to an increase in the electrical conductivity of the membrane dope, which may increase the solution’s polarity during electrospinning and improves the process, resulting in finer nanofibers.

**Figure 9:**
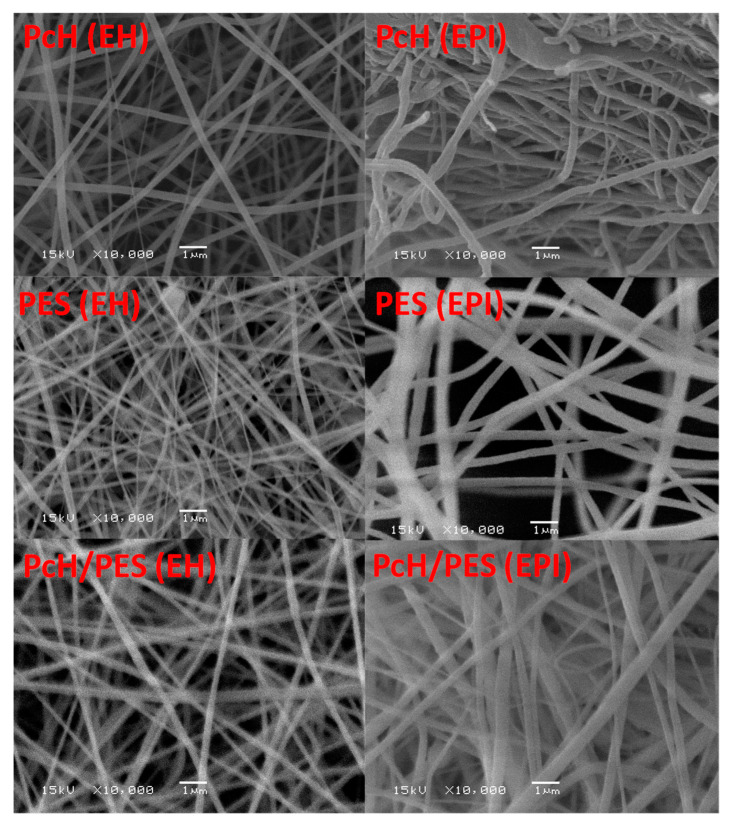
SEM imaging of pure PcH, pure PES, and PcH/PES blend membranes post-treated by heating at 60 °C (EH) and post-treated by phase inversion at 40 °C. ×10,000 was used.

The SEM surface images shown in Figure 9 illustrate different prepared membranes, in general. Almost uniform fiber diameters and smooth surfaces were fabricated. The SEM images of the same electrospinning membrane post-treated by different techniques showed a different microstructure—a more open structure with aligned fibers formed by heating at 60 °C. The post-treatment by phase inversion at 40 °C showed the following differences: 

The same PcH pure membrane became less porous, and a slightly increased roughness was observed, whereas the same PES pure membrane became more porous, and a slightly increased roughness was also determined. The shown microstructure of the pure polymers supported the determined porosity values (i.e., pure PES increased by 8.1%, and pure PcH decreased by 1.6% compared to the porosity values of the same membrane post-treated by heating at 60 °C). 

The SEM images of PcH/PES blend membrane showed a microporous structure composed of fully interconnected multifibrous layers and interstices between ultrafine fibers, with an approximate diameter of 0.19–0.44 µm.

In the phase inversion fabrication technique, the skin layer formed immediately from the casted film when the nascent membrane was immersed into the coagulation bath, and then the support layer formed because of the double diffusion of the solvent and coagulant through the skin layer. A previous work [62] reported that the increase in the coagulation bath temperature had little influence on the PVDF membrane phase separation rate because of the slow interaction between water and PVDF. On the other hand, at a high coagulation bath temperature, crystallization can be suppressed and liquid-liquid demixing can take place before crystallization [63].

**Figure 10:**
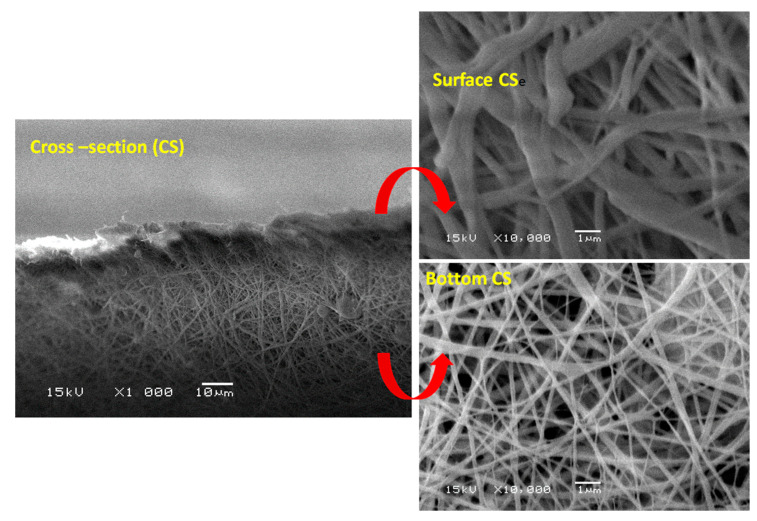
SEM cross-section imaging of PcH/PES blend membranes post-treated by phase inversion at 40 °C. ×1000 and ×10,000 were used.

Figure 10 shows SEM cross-section imaging of PcH/PES blend membranes post-treated by phase inversion at 40 °C, which confirms that the porosity at the outer surface was lower than that at the inner surface. This is due to the polymer concentration increase brought about by the solvent evaporation from the outer surface during the phase inversion process. As clearly shown in the bottom cross-section, an interconnected cellular structure, most probably brought about by the liquid-liquid phase separation mechanism, was formed in a more open structure layer. So, the different porosity may be attributed to different phase separation mechanisms.

ImageJ software was used to estimate the average fiber diameter presented in Table 2. The fiber diameters in one membrane were different with different post-treated techniques; the average fiber diameter of the membrane post-treated by heating at 60°C was lower compared to the fiber diameter of the electrospun fibrous membrane post-treated by phase inversion at 40 °C. An obvious interconnected open structure and the continuous overlapping of the electrospun nanofibers of pure PcH, pure PES, and PcH/PES blend membranes was shown. The pure polymeric fibers had a homogeneous size distribution, with a diameter of around 0.18 ± 0.03 and 0.21 ± 0.02 µm for the PES and PcH membranes post-treaded by heating at 60 °C, respectively. The same membrane showed a diameter of around 0.41 ± 0.01 µm and 0.31 ± 0.03 µm for the PES and PcH membranes post-treaded by phase inversion at 40 °C, respectively.

### 3.7. Membrane Characterization (Bulk Properties)

**Figure 11:**
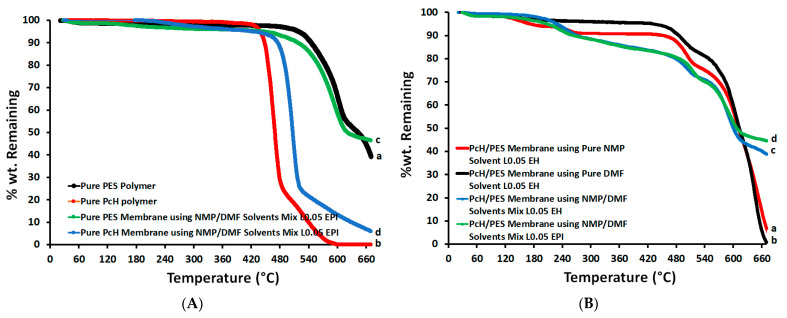
TGA of pure polymers (**A**) and blend PcH/PES membranes (**B**) using pure and/or mixed solvents.

Figure 11 shows the TGA analyses for both the PES and PcH polymers as well as for PcH/PES blend membranes using the MNP/DMF solvents mix with LiCl additive. The initial weight loss below 200 °C corresponded to the removal of moisture and/or solvent; this was less than 2–8%. The onset of the initial decomposition of the backbone of the PcH polymer was around 400 °C. This temperature was reduced to around 210 °C for the pure PcH film that was prepared using the NMP/DMF solvents mix. The second thermal degradation step was started around 480 °C. The weight loss of the PES membrane was around 450 °C, which can be attributed to sulfur dioxide cleavage and ether bond cleavage. At higher temperatures, the second thermal degradation stage started around 610 °C, and the backbone (benzene ring) decomposed. This temperature was reduced to around 430 °C for pure PES film prepared using the NMP/DMF solvents mix. It was observed that the PES membrane is more thermally stable than the PcH film prepared using the NMP/DMF solvents mix. For example, %wt remaining are 53.1 and 12.1% for PES and PcH, respectively, at 610 °C. Figure 11B proposes that the PcH/PES blend using the NMP/DMF solvents mix is phase-separated and incompatible at the molecular level, but the PcH/PES blend prepared using the NMP/DMF solvents mix is more thermally stable than the same blend using pure individual solvents.

**Figure 12:**
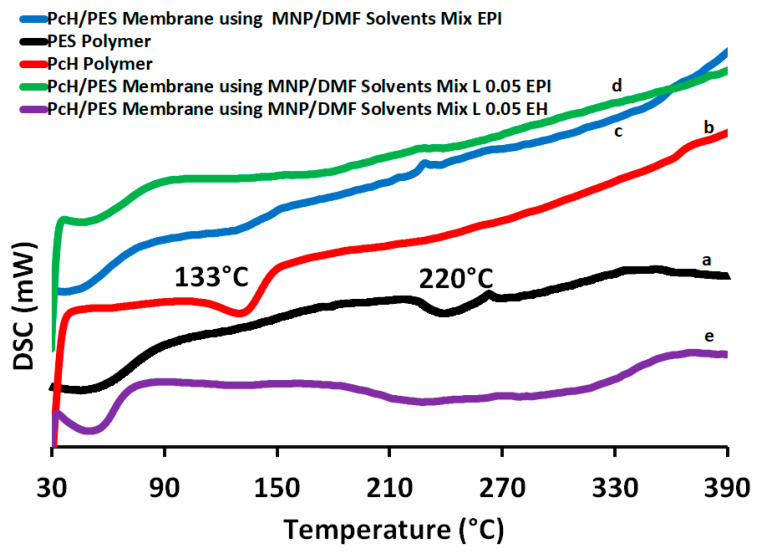
Differential scanning calorimeter (DSC) analysis of PES polymer (**a**), PcH polymer (**b**) PcH/PES blend membrane using a NMP/DMF solvents mix without LiCl post-treated by heating at 60 °C (**c**) and with LiCl post-treated by phase-inversion at 40 °C (**d**) and by heating at 60 °C (**e**).

Figure 12 shows the DSC heating scans for both the PES and PcH polymers as well as for the PcH/PES blend membranes using the MNP/DMF solvents mix with absence and presence the LiCl additive. The PcH polymer shows a melting peak at 133 °C [64], which is due to the melting of the crystalline phase of the polymer. The differences observed in the shape of the peaks obtained from the prepared membranes are attributed to the existence of more defective crystals and/or the different crystalline phases present in the PcH polymer and/or the blending with the PES polymer [65]. The DSC analysis revealed that the glass transition temperature (T_g_: onset temperature) for the PES polymer was 228 °C, and decreased only very slightly upon the blend membrane formation (around 200 °C), as in miscible blends the T_g_ of the resultant mixture usually falls in between the parent polymers.

**Figure 13:**
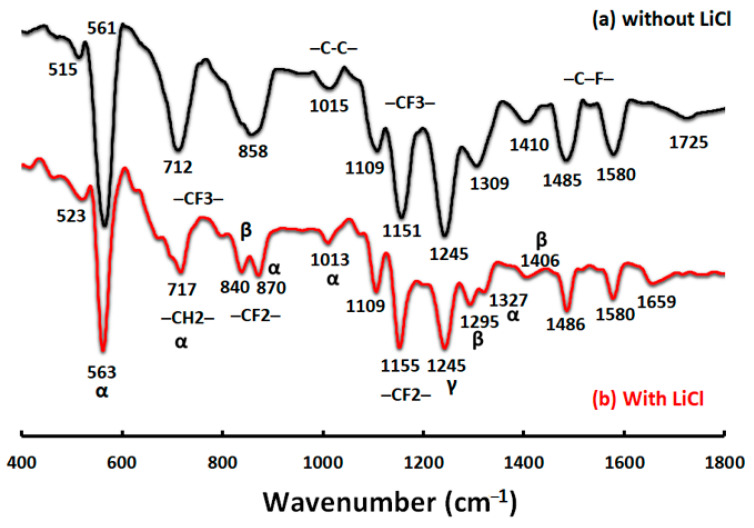
Fourier transfer infrared spectroscopy-attenuated total reflectance (FTIR-ATR) analyses of blend PcH/PES membranes using a NMP/DMF solvents mix without (**a**) and with (**b**) LiCl additive.

The FTIR-ATR results were assigned to the top surface of the prepared blend membranes using the two post-treatment techniques (Figure 13). The depth of penetration for the membrane surface was about 0.44–0.95 µm. The porous membrane (surface) possessed a very large and irregular air/solid interface, which greatly scattered the photons (especially for the wavenumbers higher than 1600 cm^−1^), causing a remarkable slope and low signal-to-noise ratio. For that, the FTIR-ATR signals are shown in Figure 13 in the range of the 1600–400 cm^−1^ wavenumber plus the signal of the OH region. The PVDF polymer has five different polymorphs: α (phase II), β (phase I), γ (phase III), δ, and ε [66]. The characteristics obtained at 1410–1580 cm^−1^ are attributed to –C-F– stretching. Moreover, the peaks shown at 870 and 1155 cm^−1^ are assigned to the –CF2– group. Likewise, the peaks obtained around 1015 cm^−1^, 840 cm^−1^ to 870, and 717 cm^−1^ correspond to the –C-C– skeletal vibration, vinylidene group, and –CH2– bonding, respectively. Therefore, all the peaks including 717, 870, and 1151 cm^−1^ can be attributed to the –CF3– group stretching. Peaks at 563, 717, 870, 1013, and around 1327 cm^−1^ are assigned to non-polar α crystals (phase II). The β crystal (phase I) is associated with absorptions at 840, 1295, and 1406 cm^−1^, whereas the peak at 1245 cm^−1^ is characteristic of the γ crystal (phase III) of the PVDF backbone of the PcH co-polymer. The α phase is normally formed at a high temperature (≥20 °C) coagulation bath, whereas thermodynamically a less stable β phase is typically formed at a room temperature coagulation bath [67,68]. The temperature favors the mobility of PcH chains and facilitates the formation of the stable non-polar α phase with a monolithic lattice structure.

The elemental composition of the pure PES and PcH membranes and the blend PCH/PES membranes investigated by EDX analysis and both At% and mass% is shown in Table 3. It was observed that the pure PES membrane contained carbon, oxygen, sulfur, and other trace elements, which may be traced from the used solvent or neat polymer. The pure PcH membrane contained carbon, a high amount of fluoride, and other trace elements, which may be traced from the used solvent or neat polymer. The detector of the used EDX does not detect the lithium element. The blend PcH/PES membranes contained carbon, oxygen, sulfur, and fluoride. The increase of sulfur content in the blended membranes may be a sign of the formation of a new blended polymer.

### 3.8. Water Vapor Permeability

The evaporation of the solvent at the outer surface of the membrane created a dense skin layer with a high concentration of the polymer near the outer surface of the membrane, and, due to that, the membrane’s water permeability decreased. The addition of LiCl to the polymer solution was effective to prepare the membrane with higher water permeability.

As shown in Table 4, in general, the Water Vapor Permeability (WVP) of pure PcH membrane is higher than the WVP of a pure PES membrane. The pure PcH prepared by the EPI technique showed slightly lower water vapor permeability than the same membrane prepared by using the EH technique, whereas the reverse is correct for the pure PES membranes. However, the PcH/PES blend membranes post-treated by phase inversion showed almost double water vapor permeability than the corresponding membrane composition post-traded by heating at 60 °C. It is possible to deduce from the results that the presence of a dense skin-layer decreases the Water Vapor Transmission Rate (WVTR). One would suspect swelling to occur for membranes prepared by the EH technique, and that swelling would cause a conformational change in the microstructure of the film, that would open up the polymer structure to allow an increase in the determined WVTR.

## 4. General Discussion

In this paper, a blend of poly(vinylidene fluoride-co-hexafluoropropylene) (PcH) and poly(ethersulfone) (PES) (18 wt.% total polymer concentration) was a successfully prepared using N-methylpyrrolidone (NMP) and N, N-Dimethylformamide (DMF) solvents mixture with a ratio of 1:9, while lithium chloride was used as an additive. The used polymers’ and solvents’ compatibility showed small differences in the overall solubility parameter, which resulted in a homogenous soluble membrane dope. The fabricated PcH/PES blend membrane was prepared by using electrospinning followed by two different post-treated techniques: heating at 60 °C and phase inversion at 40 °C and 60 °C.

The polymer and solvent compatibility, the overall solubility parameter, the dielectric constant point, the boiling points, and surface tension of the used solvents, etc., affect the relative evaporation rate of each solvent and consequently the ratio of the presence of the (polymer) components on the membrane surface and/or membrane microstructure. All the mentioned parameters should be considered carefully, as well as adding LiCl to the blend polymers that enhanced the electrospinning process, which resulted in free-bead fiber formation.

Lithium Chloride (LiCl) improves the electrical conductivity of the membrane dope, which was reflected in the solution polarity during electrospinning and positively affects the electrospinning process. However, an excessive LiCl concentration seems to have a negative effect on the Rayleigh instability and increases the bending instability of the produced fibers, which facilities the formation of 3D fiber lumps. For that reason, the lowest concentration of LiCl was used to complete this work. The effect of LiCl concentration on the static water contact angle of the prepared membranes using both preparation techniques, EH and EPI, is not significant. However, the increases in LiCl concentration seem to have a negative effect on the membrane porosity. Without LiCl additive, a completely different membrane morphology (i.e., beads-rich structure) was formed, that is thick (0.11 ± 0.01 and 0.13 ± 0.01µm) and possesses a high surface roughness (3.4 ± 0.15 and 2.9 ± 0.05 µm), the highest static water contact angle (145.6° ± 6.4 and 151.8° ± 4.9), and the lowest porosity (65.0° ± 8 and 70.7° ± 7) for the EH and EPI preparation techniques, respectively.

An innovative method of mixing electrospinning and phase inversion presented a unique membrane microstructure and properties. The co-effect of the rate of solvents evaporation and the coagulation water entry and its syringe effect on the porosity and surface roughness of the produced membranes was recognized. Moreover, adding the PcH polymer to PES by 4 wt.% resulted in an increase in the membrane porosity. This can be attributed to the formation of a less viscous membrane dope than the pure PES, which facilitates the demixing process and the higher porosity. However, an extra increase in the PcH polymer concentration, higher than 4%, did not affect the static water contact angle in the case of the EPI technique. For that reason, a PcH polymer concentration higher than 4% will result in an unnecessary increase in the membrane’s cost without a significant effect on the membrane properties. The obtained results suggest that the fiber diameters were mainly affected by the post-treated technique rather than the membrane composition and/or the electrospinning process. The membrane structure formation mechanism in the case of using the phase inversion post-treatment most probably changed from polymer crystallization to liquid-liquid phase separation with the formation of a skin-dense layer on top of a more open structure. A higher cooling rate means shorter coarsening time, which produces a lower porosity and lower water permeability.

The increase in the coagulation bath temperature resulted in a decrease in the membrane porosity (up to 23.3% reduction). However, the used solvents are phase-separated and incompatible at the molecular level, as illustrated by TGA analysis. The membrane bulk properties were enhanced by using the phase inversion post-treatment, as well as the solvent evaporation, which was complete to avoid toxicity when these membranes are used. Although, the temperature of the coagulation bath favors the mobility of PcH chains and facilitates the formation of the stable non-polar α phase (phase II), β crystal (phase I) and γ crystal (phase III) were determined in the PVDF backbone of the PcH co-polymer.

With the presence of the plastic net support, whose structure is well filled by the polymer mix and thus affects the determined thickness of the formed membranes, the membrane thickness of the formed membranes can be controlled depending on the electrospinning duration. The EDX analysis indicated the presence of both sulfur and fluoride in the composition of the produced novel blend membrane. The membrane post-treated by using phase inversion of nanofibers showed an improvement in water gas flux. The novel PcH/PES blend membranes post-treated by phase inversion showed almost double WVP (4.37 × 10^−5^ g·m/m^2^·day·Pa) than the corresponding membrane composition post-traded by heating at 60 °C (2.91 × 10^−5^ g·m/m^2^·day·Pa) and higher than the WVP (3.62 × 10^−5^ g·m/m^2^·day·Pa) of the pure PcH membrane post-treated by phase inversion.

## 5. Conclusions

In this work, an innovated blend membrane using a lower percentage of an expensive polymer, 4 wt.% PcH, and a higher percentage of a cheap polymer, 14 wt.% PES, by using an innovated EPI technique, was successfully presented and studied. SEM images of the same electrospinning membrane post-treated by different techniques, EH and EPI, showed different microstructures. In particular, the novel developed membrane, by using electrospinning followed by phase inversion technique, EPI, has shown good efficiency in terms of properties and features as a membrane suitable for membrane distillation (MD). A novel PcH/PES blend membrane that has high porosity (84.4% ± 0.6), a hydrophobic surface (136.39° ± 3.1 static water contact angle), and a water vapor permeability around 4.37 × 10^−5^ g·m/m^2^·day·Pa was successfully presented.

By simple calculation, the fabricated PcH/PES blend membrane that includes about 4 wt.% PcH showed a higher WVP than the pure PcH polymer membrane (of 18 wt.% PcH) that resulted in a reduction in the membrane cost of over 50% relative to the pure PcH membrane, in which an economic value for the presented novel blend membrane is pronounced.

Testing the produced novel membranes using a MD system is the aim of the next step in the near future, and the required system is now under construction. Numerical models are needed to accurately predict the optimum working conditions to achieve the highest yield and efficiency. In addition, membrane fouling due to hazardous and polluted water should be studied in the future.

## Figures and Tables

**Table 1:** Coded name and composition of the fabricated membranes.

NO.	Membrane Code	Solvent(s) Type	PES Concn. (wt.%)	PcH Concn. (wt.%)	LiCl Concn. (wt.%)
1	144 NMP	NMP	14	4	0
2	144 DMF	NMF	14	4	0
3	18019 PES EH and EPI	1 NMP:9 DMF	18	0	0
4	01819 PcH EH and EPI	1 NMP:9 DMF	0	18	0
5	18019 L_0.05_ PES EH and EPI	1 NMP:9 DMF	18	0	0.05
6	01819 L_0.05_ PcH EH and EPI	1 NMP:9 DMF	0	18	0.05
7	14419 EH and EPI	1 NMP:9 DMF	14	4	0
8	14419 L_0.05_ EH and EPI	1 NMP:9 DMF	14	4	0.05
9	14419 L_0.1_ EH and EPI	1 NMP:9 DMF	14	4	0.1
10	14419 L_0.5_ EH and EPI	1 NMP:9 DMF	14	4	0.5
11	10819 L_0.05_ EH and EPI	1 NMP:9 DMF	10	8	0.05
12	41219 L_0.05_ EH and EPI	1 NMP:9 DMF	4	12	0.05
13	14419 L_0.05_ EPI at 60 °C	1 NMP:9 DMF	14	4	0.05
14	14419 L_0.05_ EPI at 80 °C	1 NMP:9 DMF	14	4	0.05

**Table 2:** Average fiber diameter (µm) of the fabricated membranes.

Membrane Code	Average Fiber Diameter (µm)
PES L_0.05_ EH	0.18 ± 0.03
PES L_0.05_ EPI	0.41 ± 0.01
PcH L_0.05_ EH	0.21 ± 0.02
PcH L_0.05_ EPI	0.31 ± 0.03
14419 L_0.05_ EH	0.22 ± 0.05
14419 L_0.05_ EPI Bottom	0.44 ± 0.04
14419 L_0.05_ EPI Top	1.49 ± 0.20

**Table 3:** Elemental composition of selected prepared pure polymer and blend membranes.

Membrane Code	18019 L_0.05_ EPI		01819 L_0.05_ EPI		14419 L_0.05_ EPI		14419 L_0.05_ EH	
Element	Mass%	At%	Mass%	At%	Mass%	At%	Mass%	At%
C	68.68	77.51	31.10	41.74	55.36	68.75	53.53	66.31
O	21.60	18.31	0.0	0.0	12.38	11.55	12.55	11.67
F	0.0	0.0	68.30	57.97	14.82	11.63	19.77	15.48
S	8.33	3.52	0.0	0.0	16.58	7.71	13.48	6.26
Others traces elements	1.39	0.66	0.6	0.28	0.87	0.36	0.67	0.28

**Table 4:** Water vapor transmission rate (g/m^2^·day) and water vapor permeability (g·m/m^2^·day·Pa) of the prepared membranes.

Membrane Code	Water Vapor Transmission Rate (g/m^2^·Day)	Water Vapor Permeability (g·m/m^2^·Day·Pa) × 10^−5^
**01819 L_0.05_ PcH EH**	1255.0	4.20
**01819 L_0.05_ PcH EPI**	1171.0	3.62
**18019 L_0.05_ PES EH**	1635.0	2.83
**18019 L_0.05_ PES EPI**	1809.7	2.34
**14419 EH**	1567.9	2.01
**14419 EPI**	1391.1	4.26
**14419 L_0.05_ EH**	1621.0	2.91
**14419 L_0.05_ EPI**	1144.8	4.37
**10819 L_0.05_ EH**	1531.0	1.02
**10819 L_0.05_ EPI**	2080.0	1.02
**14419 L_0.05_ EPI at 60 °C**	1206.4	3.70
**14419 L_0.05_ EPI at 80 °C**	1410.9	2.88

## Data Availability

Data is contained within the article.
